# Case report: A case of spinal muscular atrophy in a preterm infant: risks and benefits of treatment

**DOI:** 10.3389/fneur.2023.1230889

**Published:** 2023-09-12

**Authors:** Elisa Nigro, Eyal Grunebaum, Binita Kamath, Christoph Licht, Caroline Malcolmson, Aamir Jeewa, Craig Campbell, Hugh McMillan, Pranesh Chakraborty, Mark Tarnopolsky, Hernan Gonorazky

**Affiliations:** ^1^Division of Neurology, The Hospital for Sick Children (SickKids), Toronto, ON, Canada; ^2^Division of Immunology, The Hospital for Sick Children (SickKids), Toronto, ON, Canada; ^3^Division of Gastroenterology, Hepatology and Nutrition, The Hospital for Sick Children (SickKids), Toronto, ON, Canada; ^4^Division of Nephrology, The Hospital for Sick Children (SickKids), Toronto, ON, Canada; ^5^Division of Hematology/Oncology, The Hospital for Sick Children (SickKids), Toronto, ON, Canada; ^6^Division of Cardiology, The Hospital for Sick Children (SickKids), Toronto, ON, Canada; ^7^Department of Pediatrics, Children's Hospital, London Health Sciences Centre, Western University, London, ON, Canada; ^8^Department of Pediatrics, Children's Hospital of Eastern Ontario Research Institute, Ottawa, ON, Canada; ^9^Department of Pediatrics, Newborn Screening Ontario, Children's Hospital of Eastern Ontario Research Institute, Ottawa, ON, Canada; ^10^Department of Pediatrics, McMaster Children's Hospital, Hamilton, ON, Canada

**Keywords:** spinal muscular atrophy, preterm, newborn screening, gene therapy, Zolgensma

## Abstract

Spinal muscular atrophy (SMA) is a neuromuscular genetic disorder caused by the loss of lower motor neurons leading to progressive muscle weakness and atrophy. With the rise of novel therapies and early diagnosis on newborn screening (NBS), the natural history of SMA has been evolving. Earlier therapeutic interventions can modify disease outcomes and improve survival. The role of treatment in infants born preterm is an important question given the importance of early intervention. In this study, we discuss the case of an infant born at 32 weeks who was diagnosed with SMA on NBS and was treated with Spinraza^®^ (Nusinersen) and Zolgensma^®^ (Onasemnogene abeparvovec-xioi) within the first 2 months of life. With the scarce evidence that currently exists, clinicians should be aware of the efficacy and safety impact of early therapy particularly in the preterm infant.

## Introduction

Spinal muscular atrophy (SMA) is a neuromuscular disease characterized by the degeneration of lower motor neurons caused predominantly by defects in the *SMN1* gene encoding the survival motor neuron protein (SMN). SMA is an autosomal recessive disorder that results in progressive muscle weakness and atrophy leading to significant morbidity and mortality. Approximately 70% of all cases are severe, with a median survival of 7 months and a mortality rate of 95% by 18 months of age (1, 2).

SMN is highly expressed during the gestation period and in the first few months of life. Between the fetal and postnatal phases, SMN protein content in the spinal cord declines 6.5-fold from its peak abundance during the embryonic period (2, 3). The loss of the lower motor neurons starts before the infant presents clinically and without any physical signs of SMA. Ramos et al. described a potential therapeutic window, encompassing the last trimester of gestation and the first 3 months after birth when SMN protein decreases rapidly (4). Given this biology, early diagnosis and intervention have a critically important impact on the natural course of the disorder. Since 2017, various novel therapies have been approved which have changed the natural history of SMA.

Newborn screening (NBS) is completed shortly after birth to identify treatable diseases in the pre-symptomatic period. SMA can be detected using NBS, allowing for early diagnosis and potential intervention prior to, or early on in symptom onset. In Ontario, Canada, NBS was first implemented in clinical settings in 2006, and SMA was included in the screening as of 2020. Since then and until this study was submitted, approximately 394,299 patients have been screened, and 24 cases have been diagnosed in Ontario (5).

The implementation of NBS has led to an increase in pre-symptomatic therapy in the management of SMA (6). However, none of these therapies have been approved to treat preterm infants nor are there any available data to suggest the optimal time to treat a preterm infant safely. This has led to the question of when is the safest time to treat a preterm infant given the physiology of SMA and the potential risk associated with these interventions.

According to the WHO, the definition of a preterm infant is a baby born <37 weeks gestation (7). The combination of NBS and uncharted territory in the preterm world has led to a pathway of uncertainty. Lee et al. provided a recommendation to not treat preterm infants with SMA until the 37th week of gestational age (GA) because concomitant treatment with corticosteroids may adversely impact neurological development (8). They examine two cases with the use of gene replacement therapy on a 34 + 6-week GA infant and another of 34 + 1 weeks GA and made this recommendation given the potential complications of gene therapy in a younger infant.

In Canada, two therapies are currently approved for newborns who are <6 months of age with two or three copies of *SMN2*. The first is nusinersen, an antisense oligonucleotide therapy. The second is a gene replacement therapy, onasemnogene abeparvovec-xioi. A third medication called risdiplam (evrysdi^®^) is approved by Health Canada for infants older than 2 months of age.

Here, we will examine the outcome of a pre-symptomatic preterm infant diagnosed with SMA through the NBS program in Ontario and review the current evidence around the risk of therapy on this pediatric population.

## Case

A 32-week-old baby boy was born via spontaneous vaginal delivery due to premature rupture of membranes after an uncomplicated pregnancy. His neonatal course was complicated by respiratory distress secondary prematurity requiring 3 days of continuous positive airway pressure (CPAP) in the neonatal intensive care unit (NICU) and phototherapy for neonatal jaundice. He was screened positive for SMA on NBS and had confirmatory genetic testing for homozygous loss of *SMN1* and two copies of *SMN2*. There was no family history of SMA or neuromuscular disease. Physical examinations at birth and during his first few weeks of life were normal, including good neurological and motor functioning. He scored 2/26 on the Hammersmith Infant Neurological Examination (HINE) at birth. He was seen by an occupational therapist on day 10 of life and was deemed safe for swallowing.

With respect to potential therapeutic interventions, his parents were counseled on all the therapies available in the treatment of SMA as well as the option to not treat with a therapy and allow for the natural course of the disease. Parents were interested in treating early and preferred to proceed with gene replacement therapy as this would be a one-time infusion. Risdiplam was not yet approved in Ontario at the time of this patient's birth but was discussed and not favored as an option to wait to treat until he was eligible. Given his premature age, there was no clear evidence to suggest that gene therapy would be safe before 39 weeks GA. As a result, we opted to administer nusinersen before 40 weeks of age due to the localized effects of the drug and less potential for systemic effects that may impact a premature infant. As such, he received three loading doses of nusinersen at 36, 38, and 40 weeks GA until he was able to be administered onasemnogene abeparvovec-xioi. He tolerated nusinersen well with no complications, and we did not observe any neurological abnormalities.

**Figure 1:**
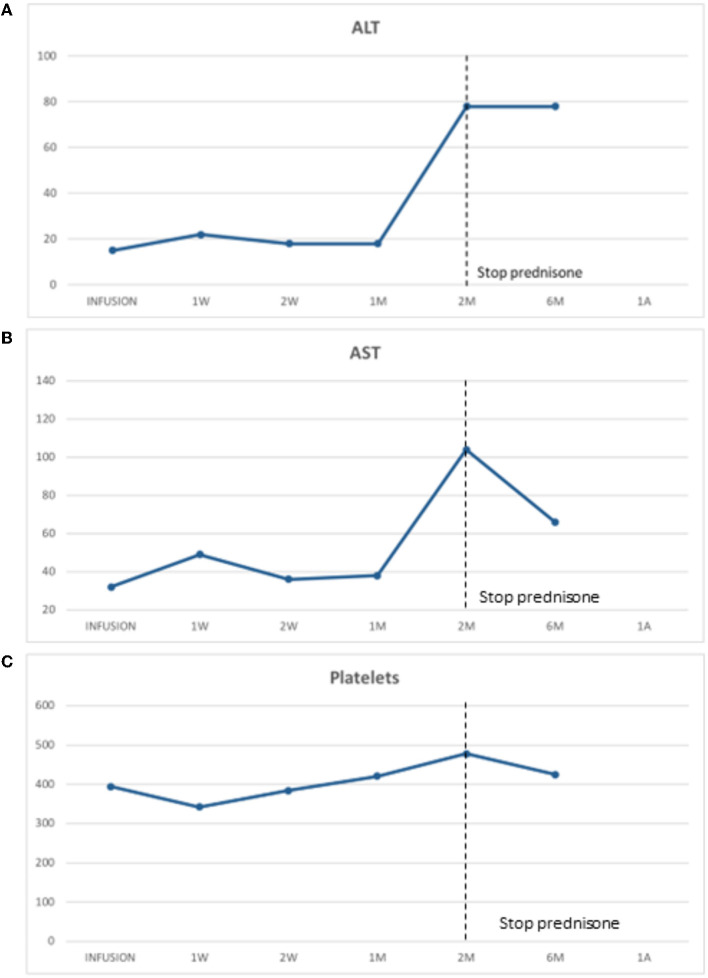
**(A)** Alanine transaminase (ALT) was stable post-gene therapy infusion during prednisone and initially elevated upon prednisone wean but later stabilized. **(B)** Aspartate transaminase (AST) was stable post-gene therapy infusion with prednisone daily. It was initially elevated and then stabilized post-prednisone weaning. **(C)** Platelet post-gene therapy infusion. There was a typical drop in platelet level post-gene therapy with a nadir at day 7 postinfusion which later recovered.

He later received onasemnogene abeparvovec-xioi at 10 weeks of age. He was monitored weekly as an outpatient for 2 months postinfusion to ensure no adverse events would occur. His baseline liver biochemical tests including serum transaminase levels and abdominal ultrasound (US) were normal. He was started on prednisone (1 mg/kg) the day before onasemnogene abeparvovec-xioi infusion and remained on prednisone at the same dose for 4 weeks. At this time, the prednisone was slowly tapered over the course of an additional 4 weeks. This was performed in conjunction with weekly bloodwork, assessing liver biochemistry and hematology and then changed to biweekly and then monthly for 6 months post-prednisone completion. At the onset of prednisone weaning, his serum transaminases were elevated but steadily improved during the remainder of the tapering period (Figures 1A, B).

**Figure 2:**
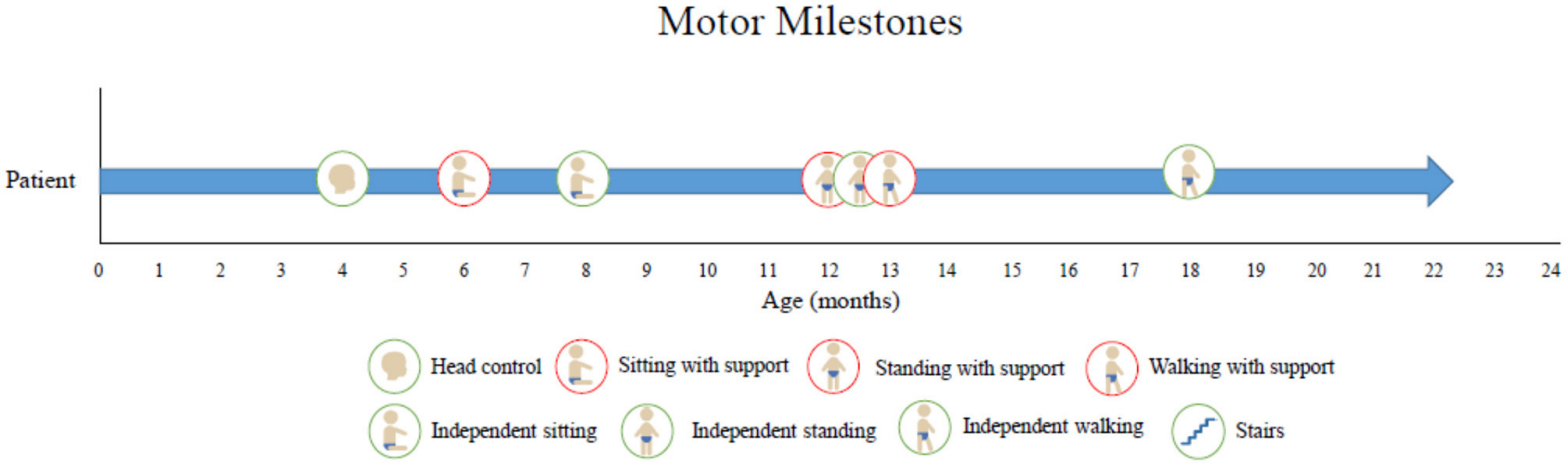
Motor milestone development. At 4 months of age, he obtained head control, at 8 months, he achieved sitting independently, and at 12 months, he was able to stand with support. Presently, at 20 months of age, he is currently walking short distances, can stand independently, reaches for objects, and has good head control.

**Figure 3:**
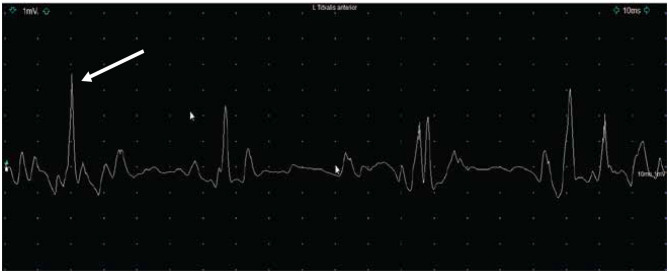
Electromyography (1mv/Div; 10 ms/Div) (EMG) with a concentric needle over the left tibialis anterior muscle showing the presence of high-amplitude motor unit potential between 2.5mV and 4mV at 20 months of age. We did not observe any spontaneous activity.

Along with our physical examinations and neurological examinations regularly, he was assessed by a physiotherapist who obtained motor scores following onasemnogene abeparvovec-xioi administration. He obtained head control at 4 months of age and achieved independent walking at 18 months both of which are delayed even for his preterm age. Presently, at 20 months of age, he is currently pulling to stand, walk, and sit independently, is reaching for objects, and has good head control (Figure 2). He is eating orally, is gaining weight, has no respiratory concerns, and can say up to 10 words. In a recent EMG study, we detected high-amplitude motor unit potentials with no signs of denervation which might suggest some degree of chronic motor neuron involvement, while nerve conduction studies were normal (Figure 3).

## Discussion

In our patient, the mild motor milestone delay plus the electrophysiological findings might suggest the requirement of initiation of treatment as soon as possible to improve the outcome.

The importance of early intervention in the diagnosis and management of infants with SMA is becoming increasingly clear to save motor neurons and prevent declines in motor functioning. Signs and symptoms of SMA often begin in the first 6 months of life (1). Given this and the potential adverse effects that novel therapies play in SMA management, impacts to a preterm infant come with many considerations where a balance of implementing early treatment while considering the physiology of a preterm infant is necessary. As mentioned before, we will be focusing on the use of gene replacement therapy in preterm infants and the currently available evidence.

### Impact of steroid use on the immune system

Currently, the use of onasemnogene abeparvovec-xioi requires the use of systemic corticosteroids (prednisone) at 1 mg/kg for at least 1-month posttherapy with the 2nd month tapering the dose to help with the management of the adverse effect of liver inflammation that comes with gene therapy. According to the FDA, onasemnogene abeparvovec-xioi is not recommended until preterm infants reach full-term gestational age because the use of corticosteroids can affect neurological development (9). However, in a study of preterm infants with bronchopulmonary dysplasia (BPD), the primary management strategy is a course of systemic corticosteroids for a short period of time (10). The primary risks are adrenal insufficiency and growth failure, but with close monitoring and recognition, these adverse effects could be managed accordingly or prevented. Similar to onasemnogene abeparvovec-xioi in preterm infants with SMA, the benefits of having the corticosteroid and recovering from the disorder have a far greater impact on the outcome of the disease than the adverse effects of corticosteroid use.

Regarding immunosuppression in a preterm infant, the immune system is not fully developed from an embryological standpoint and is not exposed to prior antigens. The use of corticosteroids in addition to adeno-associated virus 9 vector (AAV9) needed in onasemnogene abeparvovec-xioi adds to the stress of the immune system. Corticosteroids lead to further immunosuppression in the preterm infant that is already weakened. Several immune pathways have reduced involvement, and immune immaturity is present in premature neonates in proportion to their age (10). Fragile skin, moderate-to-severe hypogammaglobulinemia, decreased lymphocyte counts, plasma complement, and antimicrobial peptide levels are also seen in premature babies (10). Extreme preterm children lack trans-placental transfer of maternal antibodies, which occurs primarily during the third trimester of pregnancy. Given the specific susceptibility of preterm newborns' developing organs, it is probable that a lower inflammatory response during fetal life protects against the potentially harmful consequences of an overactive immune system (10). Most of the adverse effects related to AAV9 expressing SMN therapy have been related to the immune response toward the viral infection. Thus, it might be that preterm infants will have an advantage at the time of receiving therapies where AAV is the vector. Moreover, AAV infection has not been directly connected to any specific disease, thus reducing the need of high-dose steroids for a prolonged period of time (10).

### Steroids and the developing brain

With the administration of onasemnogene abeparvovec-xioi, infants are started on low-dose prednisone for the management of liver inflammation post-onasemnogene abeparvovec-xioi. There is growing concern and evidence that the use of corticosteroids can impact the developing brain in a preterm infant. Most of the evidence of the impact of corticosteroids on neurodevelopment comes from the use of steroids in chronic lung disease, and the risk of short-term corticosteroid use outweighs the potential benefits in particular when considering SMA (8). An example is in the treatment of BPD, where infants are given dexamethasone for earlier weaning and extubation of mechanical ventilation. However, studies have now shown that the administration of dexamethasone within the 1st week of life has been associated with an increased risk for adverse effects including speech, cognitive, and learning impairments (11). This is concerning when these infants are on steroids post-onasemnogene abeparvovec-xioi for at least 1 month before they are weaned off, and these infants are often still in their first 3 months of life. In a multicenter cohort study examining the use of steroids on preterm infants with BPD, infants given prednisone or methylprednisolone had less impact on the developing brain compared with infants given dexamethasone in neurodevelopment (12). This suggests that considering an alternative systemic corticosteroid may be a solution to minimizing the risk of neurodevelopmental health. Further studies are required to continue to evaluate this risk in particular preterm infants post-onasemnogene abeparvovec-xioi.

### Pediatric liver and early steroid use

The liver continues to mature right after birth regardless of gestational age, and full liver maturity takes up to 2 years to be achieved (6). Although this liver immaturity has little effect on a healthy full-term infant, preterm infants are particularly susceptible to the effect of an immature liver (6, 13). Preterm infants have a higher risk of bleeding, cholestasis, hypoglycemia, and impaired drug metabolism. This can lead to significant concerns with the metabolism of onasemnogene abeparvovec-xioi as it is encapsulated in an AAV9 vector that has been shown to cause liver inflammation and chronically elevated transaminases in patients with SMA post-gene therapy (14). Liver insufficiency is a major concern post-gene therapy administration, and onasemnogene abeparvovec-xioi has a black box warning for the potential to cause acute serious liver injury and acute liver failure (9). There have also been reported two deaths in children following onasemnogene abeparvovec-xioi administration due to liver failure (9).

In addition to liver immaturity, preterm infants are 80% more likely to have neonatal jaundice. This is a result of increased red cell breakdown and decreased bilirubin excretion and leads to unconjugated hyperbilirubinemia. There is currently no contraindication or safety data of onasemnogene abeparvovec-xioi administration and neonatal jaundice, but in a preterm infant who is at increased risk with a longer time to recover from jaundice, it is uncertain if this would be a reason to delay gene therapy.

With weekly bloodwork, the impact on the liver was assessed in our preterm infant regularly, and this allowed for early recognition of liver injury. His neonatal jaundice had resolved prior to the administration of onasemnogene abeparvovec-xioi, therefore this did not place him at higher risk.

### Pediatric nephrogenesis

Nephrogenesis is completed by 34–36 weeks of gestation, and kidney function continues to mature during the postnatal period (15). Thus, a preterm infant whose kidney development and function are delayed and immature at birth poses particular susceptibility in the preterm infant to kidney injury and long-term impacts on kidney functioning (16). This is particularly concerning in preterm infants' post-onasemnogene abeparvovec-xioi as cases of thrombotic microangiopathy (TMA) were reported post-onasemnogene abeparvovec-xioi that have led to kidney failure (9). Post onasemnogene abeparvovec-xioi preterm infants may be especially susceptible to any possible exogenous disturbance causing fluid and electrolyte management to be more challenging.

### Pediatric thrombocytopenia

Platelet count is an important consideration for infants receiving onasemnogene abeparvovec-xioi since thrombocytopenia and TMA have been previously reported in patients who received gene replacement therapy (9). Premature infants have a higher incidence of thrombocytopenia often due to multiple risk factors including small for gestational age, maternal preeclampsia, infection, and disseminated intravascular coagulation (17). Thrombocytopenia in neonates may also relate to an inability of platelet precursors or megakaryocytes to increase in size in response to increased platelet demands or thrombopoietin stimulation (18). This, therefore, decreases the ability of the neonatal hematopoietic system to respond to the need for platelets in a situation of consumption or destruction as seen with TMA or infection, which can be particularly challenging for the premature infant.

In our patient, we observed a typical decrease in platelet levels with a nadir at day 7 postinfusion which later recovered (Figure 1C). It is not clear which patients will naturally improve their platelet levels or which ones might be heading to an episode of TMA. Further investigations and better guidelines are needed in future to clarify this important question.

### Cardiac involvement in the pediatric infant

Due to cardiac immaturity, preterm newborns are subject to myocardial structural and functional maladaptation after delivery (3). The elevation of troponin I has been reported in patients post-onasemnogene abeparvovec-xioi; however, no clinically significant cardiac events have been reported (9). Animal studies have shown cardiac involvement in mice following intravenous injection of onasemnogene abeparvovec-xioi, including mild mononuclear infiltration, mild fibrosis, and scattered cardiomyocytes degeneration and regeneration (19). The use of corticosteroids in the preterm infant can also lead to thickening or hypertrophy of the left ventricle (20). This is a common side effect of corticosteroid that was reported in premature infants that were on the medication for as long as 42 days or as short as 3 days (21). It is thus recommended that preterm infants are closely monitored for cardiac involvement secondary to steroid exposure until they are discontinued. Included in our weekly bloodwork was troponin I levels which remained stable in our preterm infant without signs of elevation post-onasemnogene abeparvovec-xioi and with prednisone.

## Conclusion

With the rise in early identification of SMA through newborn screening programs, more infants are diagnosed with SMA prior to symptoms appearing allowing for clinicians to initiate treatment with a novel therapy early in the course of the disease leading to improved outcomes and saving motor neurons. With this early identification, there is a rise in identifying preterm infants who may be impacted by SMA. Although their physiologic immaturity, increased risk of gene therapy, and corticosteroid use post-gene therapy pose an increased risk to the preterm infant, waiting to treat until the infant is 39-week gestation also poses a risk of losing motor neurons and leads to symptoms. Knowing that time is a muscle and with careful monitoring and guidance from a multidisciplinary team, it is valuable to consider early treatment even in a preterm infant. Opting to bridge with nusinersen administration as a local impact rather than the systemic effects seen in onasemnogene abeparvovec-xioi may be one method to ensuring the best outcome. It is also imperative to consider that each individual case may differ, and thus, evaluating each clinical presentation on a case-by-case basis may also be warranted until there are enough data to suggest otherwise. We recognize that this is one case of a preterm infant and to investigate this further, we would require more data to suggest the risk and benefits of early treatment in the preterm infant. We hope to bring awareness to this clinical predicament and acknowledge the advent of medicine and change in the natural history of SMA with the rise of novel therapies, as well as the need to still treat the preterm infant early is imperative for the best possible outcome of the disease.

## Data availability statement

The raw data supporting the conclusions of this article will be made available by the authors, without undue reservation.

## Ethics statement

The studies involving humans were approved by the SickKids Hospital Ethics Committee. The studies were conducted in accordance with the local legislation and institutional requirements. Written informed consent for participation was not required from the participants or the participants' legal guardians/next of kin in accordance with the national legislation and institutional requirements. Written informed consent was obtained from the minor(s)' legal guardian/next of kin for the publication of any potentially identifiable images or data included in this article. Written informed consent was obtained from the participant/patient(s) for the publication of this case report.

## Author contributions

EN and HG drafted the manuscript and analyzed the data. All other authors reviewed their respective specialties and revised it critically for publication. All authors contributed to the article and approved the submitted version.
